# Testes Sorológicos para Doença de Chagas: Outra Evidência de Enigma em Doença Amplamente Negligenciada

**DOI:** 10.36660/abc.20200656

**Published:** 2020-12-01

**Authors:** André Schmidt, José Antonio Marin

**Affiliations:** 1 Universidade de São Paulo Faculdade de Medicina de Ribeirão Preto Centro de Cardiologia Ribeirão PretoSP Brasil Universidade de São Paulo Faculdade de Medicina de Ribeirão Preto - Centro de Cardiologia, Ribeirão Preto, SP – Brasil

**Keywords:** Doença de Chagas, Epidemiologia, Testes Sorológicos, Intervenção Coronária Percutânea/métodos

Cardiologistas e Gastroenterologistas atuando em ambientes variados da Faculdade de Medicina da USP em Ribeirão Preto frequentemente deparam-se com pacientes apresentando características epidemiológicas, clínicas e laboratoriais sugestivas da doença de Chagas (DC), mas os testes sorológicos específicos são negativos.^1^ Em série de 65 pacientes estudados de 01/07/2011 a 31/12/2012 com coronariografia invasiva para esclarecer o sintoma cardinal de dor precordial, todos com alterações de mobilidade segmentar ventricular (incluindo 28 com o típico aneurisma apical na ventriculografia de contraste radiológico) a sorologia com dois testes foi positiva em apenas 11(17%) dos casos.^2^ Como elucidar esse enigma, e como conduzir tais casos? A resposta talvez esteja, em parte, na experiência com esta entidade, e no conhecimento e convicção sobre a acuidade diagnóstica dos testes sorológicos empregados.

Mas o problema da questão se inicia com a assimetria conceitual que paira sobre a incerteza envolvendo o diagnóstico rotineiro de DC pela verificação sorológica de anticorpos antiparasitários. Assim, de acordo com diretrizes aceitas,^3 , 4^ enquanto um teste negativo seria suficiente para afastar o diagnóstico (inclusive autorizando doação de sangue ou órgãos sólidos), a necessidade de dois testes sorológicos positivos com métodos distintos para confirmação diagnóstica da DC foi há muito reconhecida e implementada em rotinas clínicas e de pesquisa; tal se justificaria devido à heterogênea acurácia (média da sensibilidade e especificidade) dos testes, ex. fixação de complemento, hemaglutinação indireta, imunofluorescência indireta e aglutinação direta).^5^ Mesmo com o surgimento da quimioluminescência baseada em ELISA, método automatizado para eliminar o componente subjetivo daqueles testes acima citados, a recomendação de utilizar dois testes distintos simultaneamente, seguido de um terceiro em caso de discordância inicial, persiste na recomendação da Organização Panamericana de Saúde.^6^ Contraditoriamente, nota técnica da Organização Mundial da Saúde recomendou a utilização de um único teste baseado em ELISA, por quimioluminescência para “screening” em bancos de sangue, apoiada na alegada muito alta sensibilidade (seria próximo de 99%), e com o intuito de reduzir custos ao realizar grande volume de testes de triagem em hemocentros e demais bancos de sangue.^7^ Contudo, há evidências bem documentadas de que tal protocolo possa ser inadequado.^8^

O estudo publicado^9^, apresenta dados oriundos de hemocentros no Estado do Ceará relativos a sorologia para DC ao longo de extenso período temporal e reaviva a evidência suscitada por algumas reflexões inquietantes:^10^

1. Considerando ser essa região endêmica, com transmissão vetorial ainda ativa pelo próprio relato dos Autores, chama atenção o elevado número de exames inconclusivos (70%) nos considerados doadores inelegíveis, e levantando a possibilidade de reação cruzada com *leishmania spp* , também endêmica na região, e que poderia ser responsável, pelo menos em parte, por resultados tão intrigantes.^11^ Mas é importante, desde já, enfatizar que pelo relato dos Autores, a inconclusividade inicial foi decorrente de um único teste ELISA que não seria nem positivo nem negativo. Isso é diferente de inconclusividade decorrente de dois testes sendo um positivo e outro negativo, situação reconhecida habitualmente em diretrizes.
2. Caracteristicamente no trabalho em pauta, a repetição do mesmo teste baseado em ELISA em segunda coleta mostrou que de 1.225 testes positivos ou inconclusivos pelo primeiro teste, em apenas 425 pacientes os resultados persistiam positivos ou inconclusivos. Isso sugere baixa reprodutibilidade, algo preocupante, e indiretamente corroborado pelo relato de que 43% dos excluídos no primeiro exame já haviam sido doadores. Assim, a negatividade no exame de triagem da doação anterior teria levado a potencial recepção de material oriundo de paciente “falso-negativo” para doença de Chagas.
3. Ainda, destes 425 pacientes dentre os 1.225 com resultados positivos (333) ou inconclusivos (92) persistindo após os dois primeiros testes baseados em ELISA na primeira e segunda amostras, foram escrutinizados outra vez nesta segunda amostra sanguínea com nova sorologia, desta feita com diferente método sorológico (imunofluorescência ou *Western blot* ), verificou-se que 305 foram negativos, 48 inconclusivos, e apenas 72 persistiram com testes positivos com base no terceiro exame sorológico.
4. Então, apenas houve concordância em 28% dos casos, cotejando-se os primeiros e os últimos testes. Afinal, os doadores assim aprovados são ou não portadores de DC? E os rejeitados como doadores, que probabilidade básica de DC possuem?

Verifica-se, portanto, nítida necessidade de aprimoramento no diagnóstico sorológico, pois a transmissão transfusional em nosso meio tem incidência incerta, por conta da paucidade de sintomas da fase aguda da DC, que muitas vezes se manifesta como quadro febril inespecífico, não incomum em pacientes hospitalizados. A transição para a forma indeterminada e a evolução ulterior para formas clínicas é tardia e pode não ser identificada se não forem feitas buscas ativas. Em tempos remotos descreveu-se a ocorrência de contaminação em 6 (25%) de 24 casos que receberam transfusão contaminada, tendo um caso desenvolvido sinais clínicos de miocardite na fase aguda.^12^ Mais recentemente, casos graves foram descritos em outros países.^13^ No que tange à transmissão associada a transplantes de órgãos sólidos infectados, com a concomitante imunossupressão, não são infrequentes manifestações graves e potencialmente letais na fase aguda.^14^ Além disso, também pode se dar reativação da doença em receptor insuspeito de portar DC, quando a ausência de um teste padrão-ouro para diagnóstico contribui para as ocorrências mencionadas.

A caracterização pela Organização Mundial da Saúde da DC como “negligenciada” adquire certamente mais força com a constatação de que, 50 anos após o estabelecimento de testes obrigatórios para doadores em nosso meio, ainda se verifica o descarte de bolsas por conta do elevado número de testes inconclusivos. Os impactos sociais e financeiros desta “inconsistência” sorológica estão para serem determinados.

De toda forma, essas duas vertentes do dilema imposto pela incerteza são imanentes ao contexto de situações enigmáticas envolvendo diagnóstico quando inexiste padrão-ouro, e a acurácia de todo teste empregado no “mundo-real” vá ser aferida em ambiente amostral diverso daquele em que o teste foi padronizado. Assim, persiste por se comprovar cabalmente a correção da suficiência de apenas um teste negativo de alta sensibilidade para excluir a DC.^15^ Além disso, mesmo o algoritmo sucessivo (teste positivo de alta sensibilidade seguido de outro, com alta especificidade) para comprovação diagnóstica^16^ parece desafiado por evidências como as agora publicadas nos ABC por pesquisadores do Ceará.
