# Supplementary material for: Testes Sorológicos para Doença de Chagas: Outra Evidência de Enigma em Doença Amplamente Negligenciada
Source: Arq Bras Cardiol. 2020 Dec 1;115(6):1092–3. [Article in Portuguese] doi: 10.36660/abc.20200656 (PMC8133718; doi:10.36660/abc.20200656)
Supplement: Translation

# Translation: Serological Tests in Chagas Disease: Another Enigmatic Evidence in a Disease Largely Neglected

**Authors:** André Schmidt, José Antonio Marin

**Affiliations:** 1 Brazil Universidade de São Paulo Faculdade de Medicina de Ribeirão Preto - Centro de Cardiologia, Ribeirão Preto, SP – Brazil

**Keywords:** Chagas Disease, Epidemiology, Sorologic Tests, Percutaneous Coronary Intervention/methods

Cardiologists and Gastroenterologists acting in distinct environments of our institution in Ribeirão Preto, not infrequently face patients with epidemiologic, clinical and laboratorial features of Chagas Disease (CD) in whom serological evaluation is negative for the disease.^1^In a series of 65 patients evaluated between 07/01/2011 and 12/31/2012 by invasive coronary angiography to elucidate the cardinal symptom of chest pain, all with wall motion abnormalities (including 28 presenting the typical apical aneurysm at contrast ventriculography), two distinct serological tests were positive in only 11(17%) patients.^2^How can we elucidate this enigma and provide guidance in this situation? The answer may be, in part, in the experience with this entity and in the knowledge and confidence in the diagnostic accuracy of the serological tests employed.

The hitch point begins with the asymmetrical concepts leading to uncertainty that involve the routine diagnostic procedures of CD by using serological verification of antiparasite antibodies. In accordance to current guidelines,^3 , 4^only one negative test is enough to exclude the diagnosis (granting blood donation or solid organ transplantation), while two distinct positive serological tests are needed to confirm CD diagnosis. This is an ancient practice in clinical and research routines, due to the heterogeneous accuracy (mean of sensibility and specificity) of the tests/ e. g. complement fixation, indirect hemagglutination, indirect immunofluorescence and direct agglutination).^5^Even after the development of chemiluminescence methods based on ELISA, capable of automated reading to avoid subjective interpretation as seen in the above-mentioned tests, the rule of two simultaneous distinct tests followed by a third one if a discordant result is obtained, remains in the latest PAHO guidelines for diagnosis of CD.^6^A conflicting technical note from the WHO recommended a single ELISA test for screening in blood banks, supported by its alleged high sensitivity (nearly 99%) and as a way of cost reduction when dealing with a high volume of screening as occurs in blood centers and blood banks.^7^Nevertheless, well-documented evidence exists suggesting that this protocol may not be entirely adequate.^8^

The study published in this issue of Arquivos Brasileiros de Cardiologia,^9^presents data from blood centers in Ceará state related to CD serological tests in an extensive period and renew the evidence leading to some unsettling reflections:^10^

1. Since it is an endemic region with vectorial transmission still present by the Authors report, the strikingly high number of inconclusive tests (70%) in those donors considered ineligible, rises the possibility of a cross reaction with *leishmania spp* , also endemic in the region, that could be responsible, at least partially, for those so intriguing results.^11^It is relevant, from now on, to emphasize that from the Authors report, the initial inconclusive result was based in only one ELISA neither positive nor negative. This is distinct from an inconclusive result of two tests, one positive and other negative, a common finding in clinical guidelines.
2. Characteristically in the present paper, the repetition of the ELISA test in a second sample in 1,225 previously positive or inconclusive results, only in 425 samples remained positive or inconclusive. This result suggests low reproducibility, a concerning result reinforced by their report that 43% of those excluded in the first test had previously donated blood. This indicates that the negative result in the first screening test led to the potential acceptance of a CD false-negative donor.
3. Furthermore, those 425 donors with positive (333) or inconclusive (92) results after two ELISA tests in two distinct samples, had their second sample submitted to another serological test (immunofluorescence or *Western blot* ). In this third test, 305 were negative, 48 inconclusive and only 72 remained positive.
4. In summary, there was a concordance of only 28% between the first and last tests. Can we state that those approved as donors are free of CD? What about those excluded as donors, what is their basic probability of having the disease?

There is clearly the necessity of improvement in serological diagnostic tests since transfusional transmission of CD in our country remains uncertain, due to the paucity of symptoms in the acute phase of CD, mainly a non-specific febrile condition commonly seen in hospitalized patients. The transition to the indeterminate form and its ulterior clinical forms occurs lately and may not be identified if not actively scrutinized. In ancient times a description of transfusional contamination in 6 (25%) of 24 recipients of one contaminated bag had only one case with clinical signs of the acute phase myocarditis.^12^Recently, severe cases have been described in other countries.^13^When occurring in solid organ transplants recipients, due to the concomitant immunosuppression, it is not rare severe and potentially lethal manifestations of the acute phase of CD.^14^In addition, reactivation of the disease may occur in an organ recipient not suspected of having CD. The absence of a gold-standard diagnostic test contributes to the above occurrences.

The definition by the World Health Organization of Chagas disease as “neglected” certainly acquires more strength by the recognition that 50 years after the establishment of mandatory tests in blood donors in our country, the number of discarded bags due to the number of inconclusive results is huge. Financial and social impact of this serological inconsistency are still to be established.

Anyway, these two pathways of the dilemma imposed by uncertainty are immanent to the context of enigmatic situations involving diagnosis when no gold-standard exists and accuracy of every test applied to the “real-world” will be assessed in a diverse environment from the one it was standardized. Therefore, it rests to be properly established the sufficiency of only one high sensibility serological test to exclude CD.^15^Besides, even a two-step algorithm (a positive high sensibility test followed by a high specificity one) to establish diagnosis^16^seems challenged by evidence now published by researches from the state of Ceará.
